# Supplementary material for: Association between Routine Laboratory Parameters and the Severity and Progression of Systemic Sclerosis
Source: J Clin Med. 2022 Aug 30;11(17):5087. doi: 10.3390/jcm11175087 (PMC9457158; doi:10.3390/jcm11175087)
Supplement: Supplementary file 1 [file jcm-11-05087-s001.zip › jcm-1825891-supplementary.pdf]

Supplemental table

Table S1. Routine laboratory parameters at baseline

	N*. With available data n (%)	SSc patients *presented as median (interquartile range)*
**White blood cells, G/L**	343 (92)	6.6 (5.5 ; 7.9)
**Hemoglobin, g/dL**	358 (96)	13.3 (12.5 ; 14.1)
**Neutrophils, G/L**	341 (91.6)	4.0 (3.1 ; 5.0)
**Monocytes, G/L**	341 (91.6)	0.6 (0.4 ; 0.7)
**Lymphocytes, G/L**	342 (91.9)	1.7 (1.2 ; 2.1)
**Eosinophils, G/L**	341 (91.6)	0.2 (0.1 ; 0.2)
**Basophils, G/L**	340 (91.4)	0.0 (0.0 ; 0.1)
**Platelets, G/L**	342 (91.9)	256.0 (216.0 ; 302.0)
**AST, UI/L**	337 (90.6)	21.0 (18.0 ; 26.0)
**ALT, UI/L**	338 (90.8)	16.0 (12.0 ; 21.0)
**GGT, UI/L**	334 (89.7)	21.0 (14.0 ; 38.0)
**ALP, UI/L**	339 (91.1)	64.0 (52.0 ; 85.0)
**Total bilirubin, mg/L**	333 (89.5)	4.0 (3.0 ; 5.0)
**Serum sodium, mmol/L**	343 (92.2)	140.0 (139.0 ; 142.0)
**Serum potassium, mmol/L**	342 (91.9)	4.2 (4.0 ; 4.4)
**Urea, g/L**	344 (92.4)	0.3 (0.3 ; 0.4)
**Creatinin, mg/L**	361 (97)	8.0 (7.0 ; 9.0)
**Albumin, g/L**	323 (86.8)	41.0 (38.8 ; 43.0)
**A1globulins, g/L**	291 (78.2)	2.9 (2.6 ; 3.2)
**A2globulins, g/L**	291 (78.2)	7.6 (6.8 ; 8.3)
**Beta globulins, g/L**	291 (78.2)	7.7 (7.1 ; 8.4)
**Gammaglobulins, g/L**	291 (78.2)	9.9 (8.2 ; 12.1)
**CRP, mg/L**	360 (96.7)	3.0 (3.0 ; 4.0)
**ESR, mm/h**	315 (84.6)	11.0 (6.0 ; 19.0)
**Uric acid, mg/L**	349 (93.8)	48.0 (39.0 ; 57.0)
**Ferritin, ng/mL**	336 (90.3)	70.5 (33.0 ; 142.0)
**NT pro BNP, ng/L**	352 (94.6)	92.0 (55.0 ; 206.0)

Values are presented as median (interquartile range [IQR]). N number. SSc : systemic sclerosis; CRP : c-reactive protein; ESR : erythrocyte sedimentation rate; ALP : alkaline phosphatase, AST : aspartate aminotransferase, ALT : alanine aminotransferase, GGT : gammaglutamyltranspeptidase; NT pro BNP : N-terminal pro-brain natriuretic peptide; A1 globulins : alpha 1 globulins; A2 globulins : Alpha2 globulins; G/L Giga/Liter.*

Table S2. Follow up data at T1 12 (9.0; 15.0) months

N*. With available data n (%)		
mRSS	372 (100)	3.0 (1.0; 6.0)
Relative progression of mRSS$	372 (100)	0.0 (-25.0; 16.7)
FVC (%)	367 (99)	105.0 (93.0; 116.0)
Relative decline of FVC$	366 (98%)	-2.0 (-5.0; 1.8)
DLCO (%)	368 (98%)	72.0 (59.0; 83.0)
Relative decline of DLCO$	366 (98%)	-1.8 (-8.3; 4.2)

Values are presented as median (interquartile range). Modified Rodnan skin score; %FVC forced vital capacity (% predicted value); % DLCO diffusing capacity for the lung of carbon monoxide (% predicted value). N Number.*

$ Relative progression of mRSS as follows: [(mRSS at T1- mRSS at T0)/ (mRSS at T0)] x 100. Relative decline of FVC and DLCO as follows: [(FVC at T1-FVC at T0)/ (FVC at T0)] x 100 and [(DLCO at T1-DLCO at T0)/ (DLCO at T0)] x 100.

Table S3. Correlation between routine laboratory parameters and modified Rodnan Skin Score (mRSS)

Biological parameters	correlation coefficient	P*
White blood cells	0.07	0.17
Monocytes	0.13	**0.013**
Neutrophils	0.07	0.22
Eosinophils	0.01	0.88
Lymphocytes	-0.02	0.71
Basophils	-0.05	0.36
Hemoglobin	-0.05	0.34
Platelets	0.05	0.35
CRP	0.08	0.14
ESR	0.01	0.89
NT pro BNP	0.07	0.19
Ferritin	-0.01	0.99
Uric acid	0.05	0.33
AST	-0.05	0.38
ALT	-0.03	0.60
ALP	0.02	0.76
GGT	0.03	0.58
Total bilirubin	-0.10	0.06
Serum sodium	-0.01	0.85
Serum potassium	0.08	0.15
Urea	0.03	0.54
Creatinin	-0.06	0.22
A1globulins	0.10	0.08
A2globulins	0.09	0.11
Betaglobulins	0.07	0.26
Gammaglobulins	0.01	0.83
Albumin	-0.13	**0.016***

CRP C reactive protein. ESR Erythrocyte Sedimentation Rate. ALP Alkaline phosphatase, AST aspartate aminotransferase, ALT alanine aminotransferase, GGT gammaglutamyltranspeptidase. NT pro BNP N-terminal pro-brain natriuretic peptide. A1 globulins alpha 1 globulins, A2 globulins Alpha2 globulins. Pvalue<0.05. ***Confirmed in multivariate analysis.***

Table S4A. Correlation between routine laboratory parameters and forced vital capacity (FVC %)

Biological parameters	correlation coefficient	P*
White blood cells	-0.17	**0.002**
Monocytes	-0.22	**<0.001**
Neutrophils	-0.18	**0.001**
Eosinophils	-0.01	0.83
Lymphocytes	0.01	0.94
Basophils	-0.04	0.41
Hemoglobin	0.04	0.43
Platelets	-0.06	0.26
CRP	-0.24	**<0.001***
ESR	-0.16	**0.006**
NT pro BNP	-0.10	0.05
Ferritin	-0.06	0.31
Uric acid	-0.02	0.78
AST	-0.01	0.93
ALT	-0.05	0.40
ALP	-0.03	0.95
GGT	-0.15	**0.006**
Total bilirubin	0.02	0.65
Serum sodium	0.08	0.16
Serum potassium	0.03	0.55
Urea	0.10	0.07
Creatinin	-0.04	0.50
A1globulins	-0.30	**<0.001**
A2globulins	-0.19	**0.001**
Betaglobulins	-0.21	**0.003***
Gammaglobulins	-0.19	**0.009***
Albumin	0.20	**0.002**

%FVC forced vital capacity (% predicted value). CRP C reactive protein. ESR Erythrocyte Sedimentation Rate. ALP Alkaline phosphatase, AST aspartate aminotransferase, ALT alanine aminotransferase, GGT gammaglutamyltranspeptidase. NT pro BNP N-terminal pro-brain natriuretic peptide. A1 globulins alpha 1 globulins, A2 globulins Alpha2 globulins. p value<0.05. **** Confirmed in multivariate analysis.***

Table S4B. Correlation between routine laboratory parameters and carbon monoxide lung diffusion capacity (DLCO%)

Biological parameters	correlation coefficient	P*
White blood cells	-0.23	**<0.001**
Monocytes	-0.23	**<0.001***
Neutrophils	-0.20	**0.002***
Eosinophils	-0.04	0.46
Lymphocytes	-0.05	0.94
Basophils	-0.04	0.48
Hemoglobin	0.25	**<0.001***
Platelets	-0.05	0.38
CRP	-0.25	**<0.001**
ESR	-0.29	**<0.001**
NT pro BNP	-0.29	**<0.001***
Ferritin	-0.02	0.76
Uric acid	-0.12	**0.022**
AST	-0.06	0.29
ALT	0.01	0.82
ALP	-0.17	**0.001**
GGT	-0.12	**0.034**
Total bilirubin	0.12	**0.025**
Serum sodium	0.06	0.29
Serum potassium	-0.04	0.47
Urea	-0.07	0.19
Creatinin	-0.10	0.05
A1globulins	-0.32	**<0.001**
A2globulins	-0.25	**<0.001**
Betaglobulins	-0.06	0.32
Gammaglobulins	-0.08	0.20
Albumin	0.32	**<0.001***

% Dlco diffusing capacity for the lung of carbon monoxide (% predicted value). CRP C reactive protein. ESR Erythrocyte Sedimentation Rate. ALP Alkaline phosphatase, AST aspartate aminotransferase, ALT alanine aminotransferase, GGT gammaglutamyltranspeptidase. NT pro BNP N-terminal pro-brain natriuretic peptide. A1 globulins alpha 1 globulins, A2 globulins Alpha2 globulins. p value<0.05. * ***Confirmed in multivariate analysis.***

Table S5. Correlation between routine laboratory parameters and relative progression of modified Rodnan Skin Score£ (mRSS)

Biological parameters	correlation coefficient	P*
White blood cells	0.13	0.16
Monocytes	0.19	**0.037**
Neutrophils	0.08	0.38
Eosinophils	-0.11	0.24
Lymphocytes	0.12	0.18
Basophils	0.09	0.35
Hemoglobin	-0.06	0.50
Platelets	-0.03	0.71
CRP	0.01	0.93
ESR	-0.01	0.99
NT pro BNP	0.24	**0.01**
Ferritin	0.02	0.82
Uric acid	0.04	0.66
AST	-0.08	0.37
ALT	-0.16	0.09
ALP	-0.12	0.19
GGT	-0.01	0.88
Total bilirubin	0.08	0.39
Serum sodium	-0.01	0.94
Serum potassium	-0.13	0.15
Urea	0.11	0.24
Creatinin	0.11	0.26
A1globulins	0.12	0.18
A2globulins	0.12	0.19
Betaglobulins	-0.05	0.61
Gammaglobulins	0.01	0.98
Albumin	0.01	0.88

£Relative progression of mRSS as follows is defined as follows: [(mRSS at T1- mRSS at T0)/ (mRSS at T0)] x 100. CRP C reactive protein. ESR Erythrocyte Sedimentation Rate. ALP Alkaline phosphatase, AST aspartate aminotransferase, ALT alanine aminotransferase, GGT gammaglutamyltranspeptidase. NT pro BNP N-terminal pro-brain natriuretic peptide. A1 globulins alpha 1 globulins, A2 globulins Alpha2 globulins; p value<0.05. **** Confirmed in multivariate analysis.***

Table S6. Correlation between routine laboratory parameters and relative progression of carbon monoxide lung diffusion capacity £ (DLCO%)

Biological parameters	Correlation coefficient	P*
White blood cells	-0.08	0.28
Monocytes	-0.12	0.12
Neutrophils	-0.08	0.31
Eosinophils	-0.09	0.26
Lymphocytes	-0.02	0.77
Basophils	-0.01	0.91
Hemoglobin	-0.03	0.66
Platelets	0.18	**0.022***
CRP	-0.01	0.99
ESR	-0.07	0.38
NT pro BNP	-0.07	0.35
Ferritin	-0.01	0.88
Uric acid	-0.13	0.09
AST	-0.05	0.51
ALT	0.03	0.72
ALP	-0.02	0.80
GGT	0.07	0.37
Total bilirubin	0.01	0.99
Serum sodium	0.11	0.15
Serum potassium	-0.14	0.08
Urea	-0.02	0.83
Creatinin	-0.02	0.80
A1globulins	0.09	0.30
A2globulins	-0.04	0.65
Betaglobulins	0.10	0.22
Gammaglobulins	0.04	0.67
Albumin	0.22	**0.007***

£Relative progression of FVC and DLCO as follows: [(DLCO at T1-DLCO at T0)/ (DLCO at T0)] x 100. CRP C reactive protein. ESR Erythrocyte Sedimentation Rate. ALP Alkaline phosphatase, AST asparate aminotransferase, ALT alanine aminotransferase, GGT gammaglutamyltranspeptidase. NT pro BNP N-terminal pro-brain natriuretic peptide. A1 globulins alpha 1 globulins, A2 globulins Alpha2 globulins. p value<0.05. ***Confirmed in multivariate analysis.***
